# Challenges in Immunotherapy Presented by the Glioblastoma Multiforme Microenvironment

**DOI:** 10.1155/2011/732413

**Published:** 2011-12-10

**Authors:** Christopher Jackson, Jacob Ruzevick, Jillian Phallen, Zineb Belcaid, Michael Lim

**Affiliations:** ^1^Department of Neurosurgery, The Johns Hopkins University School of Medicine, Baltimore, MD 21287, USA; ^2^Department of Oncology, The Johns Hopkins University School of Medicine, Baltimore, MD 21287, USA

## Abstract

Glioblastoma multiforme (GBM) is the most common and aggressive primary brain tumor in adults. Despite intensive treatment, the prognosis for patients with GBM remains grim with a median survival of only 14.6 months. Immunotherapy has emerged as a promising approach for treating many cancers and affords the advantages of cellular-level specificity and the potential to generate durable immune surveillance. The complexity of the tumor microenvironment poses a significant challenge to the development of immunotherapy for GBM, as multiple signaling pathways, cytokines, and cell types are intricately coordinated to generate an immunosuppressive milieu. The development of new immunotherapy approaches frequently uncovers new mechanisms of tumor-mediated immunosuppression. In this review, we discuss many of the current approaches to immunotherapy and focus on the challenges presented by the tumor microenvironment.

## 1. Introduction

Glioblastoma multiforme (GBM) (WHO grade IV astrocytoma) is the most common and malignant primary brain tumor in adults. Despite aggressive, multimodal treatment with maximal surgical resection followed by temozolomide and radiation, the prognosis for patients with GBM remains grim with a median survival of 14.6 months and a 3-year survival rate of only 10% [1]. One formidable challenge in advancing GBM therapy is the complexity of the GBM microenvironment [2]. Elucidating the details of GBM resistance to traditional therapies requires consideration not only of the intrinsic properties of tumor cells, but also how these cells interact with neural precursor cells, tumor stem cells, vascular endothelial cells, stromal cells, astrocytes, microglia, lymphocytes, extracellular matrix proteins, and cytokines. It is this dynamic interplay among diverse cell populations, cytokines, and extracellular matrix proteins that coordinates GBM tumorigenesis, growth, and invasion. Effective therapies, therefore, must not only be directly cytotoxic to a molecularly diverse population of tumor cells [3], but must also overcome the protumorigenic properties of the GBM microenvironment.

Immunotherapy is a particularly attractive approach to cancer treatment as it affords the advantages of cellular level specificity and the potential for generating long-term immune surveillance against cancer cells. The notion of activating the immune system against cancer has been around for decades but has recently come to the forefront with the FDA approval of the first therapeutic cancer vaccine for the treatment of metastatic, castration-resistant prostate cancer [4]. More recently, ipilimumab, an anti-CTLA-4 antibody, was approved by the FDA for first- and second-line treatment of unresectable or metastatic melanoma [5]. Preclinical research is rapidly identifying new immunological targets leading the way for the development of powerful combination therapies [6]. In addition, several immunotherapies are currently in clinical trials and many are producing encouraging results in a variety of cancers [7]. 

Immunotherapy for neoplasms of the central nervous system (CNS) has been hampered by the traditional belief that the CNS is immunologically privileged [8]. This theory was based on reports of a paucity of native antigen-presenting cells (APCs) in the CNS, the lack of a traditional lymphatic system, impermeability of the blood-brain barrier (BBB) to antibodies and lymphocytes [9], low baseline levels of major histocompatibility complex (MHC) expression [10], altered expression of T cell costimulatory molecules [11], and the observation that tissues engrafted into the CNS are rejected more slowly than those grafted to other sites [12, 13]. Each of these perceived impediments to immunotherapy has subsequently undergone major revisions. Microglia [14], macrophages, and dendritic cells [15, 16] act as powerful APCs in the CNS. Antigens originating within the CNS drain in the cerebrospinal fluid through Virchow-Robin perivascular spaces to nasal and cervical lymph nodes where they can be accessed by naïve T cells [17, 18]. Subpopulations of activated T cells expressing integrins, which impart CNS tropism, such as *α*4*β*7, traverse the BBB [19] where they can act as cytotoxic or helper T cells based on CD8 or CD4 expression, respectively [20]. There is also evidence to suggest that naïve T cells traffic to the CNS, especially when inflammation locally increases the permeability of the BBB [21]. Furthermore, antibodies have been isolated from the brain, albeit in much lower concentrations than in plasma [22, 23]. Taken together, these findings represent an evolution in our understanding of the interactions between the CNS and the immune system. 

**Figure 1:**
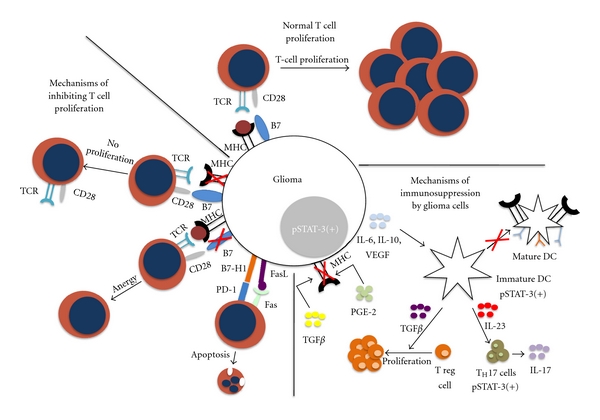
Normal T cell proliferation and mechanisms of glioma cell immunoresistance. (From top moving clockwise) *Normal T cell proliferation:* tumor cell antigens are presented by MHC and costimulatory molecules. *Mechanisms of immunosuppression:* glioma cells secrete factors leading to an immunosuppressive tumor microenvironment. TGFB and PGE-2 downregulate the expression of MHC, restricting antigen presentation and T cell proliferation. IL-6. IL-10 and VEGF are potent STAT-3 activators, leading to the proliferation of immature DCs that are not able to function as APCs. These immature DCs also secrete TGFB which aid in the proliferation of immunosuppressive Treg cells and STAT-3 positive TH17 cells. *Mechanisms of inhibiting T cell proliferation:* glioma cells downregulate MHC on their surface leading to the decreased antigen presentation and decreased T cell proliferation. Downregulation of B7 works via a similar mechanism in that the costimulatory signal is lost preventing T cell proliferation. Increased expression of B7-H1 and FasL act as proapoptotic signals for T cells.

This paradigm shift has generated enthusiasm for a potential role for immunotherapy in GBM. Despite encouraging results in rodent models, however, clinical trials of immunotherapy for GBM have been largely disappointing to date. One of the primary impediments to developing effective immunotherapies is the aforementioned complexity of the GBM microenvironment (Figure 1). Immunosuppressive cytokines such as prostaglandin E2 (PGE-2), TGF-*β*, and IL-10 are known to be highly expressed in GBMs [24, 25]. In addition, tumor-infiltrating T cells have been shown to exhibit an enriched population of CD4+, CD25+, FoxP3+ regulatory T cells (Tregs) [26]. Expression of the signal transducer and activator of transcription 3 (STAT3) is upregulated in GBM and is believed to promote immunosupression and serve as a point of convergence for several protumorigenic pathways [27]. Furthermore, tumor stem cells have been shown to be immunosuppressive in GBM [28]. Immune checkpoints, such as programmed cell death 1 (PD-1) and Cytotoxic T-Lymphocyte Antigen 4 (CTLA-4) may also be manipulated by GBM to induce T cell exhaustion [26, 29]. Finally, there is evidence to suggest that the GBM microenvironment may divert CD4+ T cell differentiation away from a tumor-directed cytotoxic Th1-mediated response and toward a Th17-mediated chronic inflammatory response [30], which has been shown to be protumorigenic in other cancers [31]. 

Identification of appropriate tumor antigens and generation of a strong antitumor immune response against such a molecularly heterogeneous neoplasm [32] poses a considerable challenge. This challenge is amplified by the immunosuppressive tumor microenvironment. Here, we review the current approaches in immunotherapy for GBM, focusing specifically on how each approach is affected by the array of challenges presented by the tumor microenvironment. 

## 2. Current Approaches

### 2.1. Cytokine Modulation

Immune responses in the CNS exhibit a distinct hierarchy skewed toward antibody responses and Th2 T cell differentiation [33–35]. It is believed that this hierarchy is maintained by the CNS cytokine milieu [35]. In the GBM microenvironment, the antitumor immune response is further suppressed by high levels of circulating immunosuppressive cytokines such as IL-10, TGF-*β*, and PGE2 as well as membrane-bound proteins such as FasL and B7-H1 (PD-L1) [36, 37]. The sources of these molecules and the details of their interactions are yet to be fully elucidated. It is clear, however, that the cytokine milieu plays a critical role in coordinating immunosupression in GBM. Clinical trials using cytokine modulation are summarized in Table 1.

#### 2.1.1. TGF-*β*

TGF-*β* is synthesized in a pre-pro-TGF-*β* form and undergoes homodimerization and cleavage by the convertase family of endopeptidases [38] to produce a C-terminal mature peptide and an N-terminal latency-associated peptide, which collectively form the small latency complex [39]. The small latency complex is then secreted from the cell and associates with specific binding proteins to form the large latency complex, which is bound by components of the extracellular matrix [39, 40]. TGF-*β* is activated when it is released from the latency-associated peptide through one of a number of context-dependent mechanisms [41]. Activated TGF-*β* regulates gene expression downstream via the SMAD family of transcription factors [39]. TGF-*β* synthesis, secretion, and signaling are reviewed in detail elsewhere [42].

TGF-*β* promotes immunosuppression in GBM by inhibiting T cell activation and proliferation, blocking IL-2 production, suppressing activity of NK cells, and promoting Treg activity [43, 44]. In addition, TGF-*β* is believed to promote tumor growth and invasion by sustaining GBM stem cells [45], promoting angiogenesis [46], and upregulating expression of molecules such as MMP-2, which are associated with tumor invasion [47]. The involvement of TGF-*β* in multiple tumorigenic pathways makes this cytokine an enticing target for immunotherapy. 

TGF-*β* expression is increased by radiation both *in vitro* [48] and *in vivo *[49]. This finding is of interest because radiation therapy is a critical component of the tripartite treatment approach of resection, temozolomide, and radiation which has become standard of care for patients with GBM [1], and because there is emerging evidence to suggest that radiation therapy may alter several components of the immune microenvironment [50–52]. Radiation-induced activation of TGF-*β* is believed to be mediated by reactive oxygen species (ROS), which have been shown to convert latent TGF-*β* preferentially to the TGF-*β*1 isoform [53]. Although this isoform plays a more minor role in GBM pathogenesis than the TGF-*β*2 isoform, available evidence suggests that TGF-*β*1 promotes immunosuppression [54] and acts as a mediator of radiation-induced DNA damage sustained by nontargeted cell populations [55]. In addition, TGF-*β*2 has been shown to increase tumor invasiveness by upregulating MMP-2 expression in glioma cells [56] and evidence from other cell lines suggests that TGF-*β*1 may be an even more powerful inducer of MMP-2 expression [57].

The results of TGF-*β* blockade in preclinical models have been generally promising. The TGF-*β*2 antisense oligonucleotide trabedersen (AP12009) has been shown to decrease tumor cell proliferation, inhibit migration, and enhance the antitumor immune response *in vitro*. A randomized, phase IIb clinical trial of trabedersen reported significantly improved tumor control and a trend toward increased 2-year survival for patients with anaplastic astrocytoma as compared with standard chemotherapy (temozolomide or a combination of procarbazine, lomustine, and vincristine) [58]. This trial did not report improved survival in patients with GBM, although a subgroup analysis of young patients with good performance status indicated a trend toward improved 2- and 3-year survival rates. Of note, the reported rate of treatment-related adverse events was approximately 20% higher with standard chemotherapy than with trabedersen. Trabedersen is currently in phase III clinical trials for anaplastic astrocytoma [59]. Understanding the role of TGF-*β* in the tumor microenvironment may have implications for standard therapies as well. For example, given that available evidence points toward a protumorigenic role for TGF-*β*, the addition of TGF-*β* blockade to adjuvant radiation therapy may prove prudent [60].

#### 2.1.2. IL-2

IL-2 is a proinflammatory cytokine which promotes T cell activation and Th1 differentiation while abrogating the immunosuppressive effects of TGF-*β* [61]. IL-2 therapy for GBM is complicated by the fact that high systemic doses of IL-2 are required to reach therapeutic concentrations in the CNS [62]. Early trials of IL-2 alone or in combination with IFN-*α* [63] or lymphokine-activated killer (LAK) cells [64] attempted to obviate the severe side effects associated with systemic high-dose IL-2 therapy by delivering IL-2 intratumorally or intraventricularly; however, the patients in these trials experienced significant adverse events resulting from local edema. A more recent trial by Colombo et al. used a retroviral vector and intratumoral implantation of retroviral-producing cells to deliver combination HSV-TK/IL-2 gene therapy followed by administration of acyclovir to 12 patients with recurrent gliomas [65]. This trial reported no major adverse events and a radiographic response rate of 50%. Evidence from preclinical models additionally suggests that IL-2 therapy generates long-lasting immune surveillance, which is capable of eliminating tumor cells both inside and outside the CNS [66]. Current approaches to IL-2 therapy for GBM are focused on combination therapy and strategies for local delivery [67].

#### 2.1.3. Interferons

Interferons are secreted by immune cells in response to viruses or other challenges and serve to coordinate the immune response. Alpha interferon (IFN-*α*), beta interferon (IFN-*β*) and gamma interferon (IFN-*γ*) have been extensively studied in cancer immunotherapy. These type 1 interferons have specifically been implicated in coordinating an antitumor immune response against GBM. A study by Fujita et al. demonstrated that mice deficient in type 1 interferons, and induced to develop gliomas *de novo* via p53 knockdown, exhibited enriched populations of tumor infiltrating myeloid-derived suppressor cells and Tregs as well as a decrease in the numbers of tumor-infiltrating CD8+ T cells [68]. Despite some preclinical evidence for efficacy against gliomas, small clinical trials using IFN-*γ* have been generally disappointing [69, 70]. Trials of IFN-*β* have produced mixed results [71–73]. The efficacy of IFN-*β* in combination with temozolomide is currently being investigated [74]. 

Of the type 1 interferons, IFN-*α* has been the most extensively studied in GBM. In a phase III study by Buckner et al., 214 patients were initially treated with BCNU and radiation. Patients with radiographically stable disease were subsequently randomized to treatment with a second course of BCNU or BCNU and IFN-*α*. This study demonstrated no difference in survival or tumor response with the addition of IFN-*α* [75]. Unfortunately, there was a significantly increased incidence of side effects, including fever, chills, myalgias, somnolence, confusion, and exacerbation of neurologic deficits in patients receiving IFN-*α*. These findings were in contrast with a prior phase II study by the same group, which reported that IFN-*α* was associated with radiographic evidence of tumor regression in 29% of patients and limited toxicity [76]. A more recent trial of IFN-*α* in combination with local BCNU delivery in patients with recurrent GBM reported 6-month progression-free survival in 2/9 patients [77]. Of interest, both patients who responded in this study were in the group receiving the lowest dose of IFN-*α*. Therefore, while grade 2 and grade 3 toxicities were observed somewhat frequently in the higher dose groups, only two grade 2 events and no events grades 3 or higher were observed in the treatment group containing the two responders.

#### 2.1.4. Miscellaneous Cytokines

Many cytokines have been evaluated for their effectiveness in GBM therapy. TNF-*α* knockout mice implanted with GL261 glioma cells have been shown to harbor a decreased number of tumor-associated macrophages and exhibit shorter survival [78]. Knowledge of the role of TNF-*α* in human gliomas, however, is limited. IL-4 has been shown to increase in CD8+ tumor-infiltrating T cells in a rat model [79]. In a small clinical trial by Okada et al., patients received vaccinations of autologous glioma cells and fibroblasts retrovirally transfected with TFG-IL4-Neo-TK [80]. Treatment was well tolerated, but there was no observed progression-free survival benefit. Locally delivered IL-12 in preclinical models increases tumor-directed T cell responses [81], improves survival, and produces variable development of durable immune surveillance [82]. Tumor stem cells secreting IL-12 have also been shown to track migrating glioma cells and prolong survival [83]. Limited evaluation of IL-12 therapy in clinical trials, however, has produced mixed results [84]. Granulocyte-macrophage colony-stimulating factor (GM-CSF) promotes a CD8+ cytotoxic T cell response when combined with antitumor vaccines [85]. GM-CSF is currently being used as an adjuvant in a phase II vaccination study of patients with newly diagnosed GBM [86]. Discovery of T cell populations producing IL-17 (Th17) [87] and their association with STAT3 expression in human cancers [88] have recently generated an interest in defining the role of these cells in GBM pathogenesis. Early preclinical studies indicate IL-17 is expressed in GBM, but the significance of IL-17 expression in the tumor microenvironment is yet to be clearly defined [30].

### 2.2. Cellular Immunotherapy

Transfer of *ex vivo* matured immune cells is showing promising results as a future immunotherapeutic intervention against malignant glioma. Initially used as a treatment for melanoma, this strategy involves infusion of autologous immune cells that were matured *ex vivo* with activity specific for glioma cell antigens. While studies have shown lymphokine-activated killer cells cannot effectively migrate across the BBB, effector T cells are able to cross the BBB allowing for a vaccine or IV strategy to be used [89]. 

#### 2.2.1. Lymphokine Activated Killer Cells

Lymphokine activated killer (LAK) cells are autologous peripheral blood lymphocytes that have been stimulated *in vitro* with IL-2 [90]. Results of early clinical trials infusing LAK cells directly into the surgical cavity showed promise for the use of LAK cells as an immunotherapeutic strategy [64, 91, 92]. The most encouraging of these early studies, Hayes et al. reported a median survival in 18 patients of 12.2 months compared with the control group of 6.2 months with minimal toxicity [93]. In 2004, Dillman et al. reported minimal toxicity and an increase in median survival in a trial of 31 patients. Median survival from the date of original diagnosis was 17.5 months versus 13.6 months for a control group of 41 contemporary GBM patients [94]. Of note, LAK cells must be administered directly to the tumor site since they fail to effectively migrate from the periphery into the brain [95]. Clinical trials using LAKs are summarized in Table 2.

#### 2.2.2. Effector T-Cells

Effector T cell therapy involves transfer of autologous cytotoxic T cells (CTLs) specific for tumor antigens, which are matured from peripheral blood mononuclear cells (PBMCs) or T cells from the tumor itself, to the host. This therapy is based on the theory that T cells can migrate to the site of a tumor by crossing the BBB, and selectively exert cytotoxic effects on tumor cells. This strategy has been studied extensively in malignant melanoma with promising results. Studies in animal models of glioma have been promising. Initial studies by Yamasaki and Kikuchi used IL-2 to activate CD8+ T cell clones with target specificity against murine malignant brain tumor cells. This strategy resulted in successful migration of T-cells to the tumor, cytotoxic activity against the tumor, and a significant increase in survival after IV infusion [96].

Early clinical studies using *ex-vivo*-expanded CTLs were largely disappointing for patients with GBM, however, more recent studies have shown promise. Tsurushima et al. reported that activating polyclonal T cells with IL-2 resulted in two patients with Grade III disease exhibiting complete tumor regression for at least 5 years with another patient having a partial regression [97]. A study using GM-CSF resulted in three of ten patients having at least partial tumor regression. All patients with a diagnosis of GBM survived at least one year from the time of adoptive transfer [98]. Another approach has been to genetically modify T cells to express a chimerical antigen receptor (CAR) for a known tumor antigen. Kahlon et al. genetically engineered CD8+ T cells to express CARs for IL-13R*α*2 and reported regression of GBM xenografts [99]. Studies in human GBM have demonstrated that CARs can migrate to tumors *in vivo* [100]. Furthermore, Ahmed et al. have shown that CARs targeted to HER2 are able to eliminate CD133+ stem cells as well as bulk tumor cells in HER2+ GBMs [101]. Clinical trials using CTLs are summarized in Table 3.

### 2.3. Antigen Identification and Targeting

Targeting of tumor-specific antigens is a promising strategy for delivering antitumor immunotherapy. The effectiveness of this approach remains controversial, however, as many vaccine trials have not demonstrated a consistent antitumor response or survival advantage despite increased tumor reactive cytotoxic T cells [102–105]. One of the challenges facing therapy directed against single antigens is the ability of a tumor to alter its antigen expression profile, resulting in immune editing. Immune editing consists of three phases: elimination, equilibrium, and escape [106]. The elimination phase is maintained by immunosurveillance of cancer cells by both the innate and adaptive immune system [107–109]. The equilibrium phase occurs when tumor cells survive the cytotoxic pressure exerted by immune cells. Finally, the escape phase results in uncontrolled tumor growth and often clinical manifestations of disease [106]. Often immune escape is preceded by mutations within cancer cells that facilitate immune evasion. For example, loss of HLA class I proteins [110, 111] and decreased response to IFN-*γ* [108, 112] have been described in adenomas of the lung and melanoma. 

Another major challenge currently limiting antigen-targeted therapies is the inability to tailor therapy to an individual tumor's antigen expression profile. The current classification scheme for glioma does not account for the molecular diversity of GBM. A new model for classification, reported by Verhaak et al., is a molecular classification of glioblastoma consisting of four clinically relevant tumor subtypes—classical, mesenchymal, proneural, and neural [113]. A comprehensive understanding of which antigens are present on each GBM subtype would allow for better targeted immunotherapy. 

#### 2.3.1. EGFRvIII

The epidermal growth factor receptor vIII (EGFRvIII) is a truncated form of the wild-type EGF receptor [114, 115] and is an attractive antigen for immunotherapy because it is not expressed by normal brain and leads to enhanced tumorigenicity of the EGFRvIII-expressing cell [116]. This truncated protein is constitutively active despite its inability to bind extracellular ligand [117]. Efforts to target EGFRvIII, however, have been significantly hampered by immune editing [106]. For example, unpublished data from CDX-110 clinical trials reported the EGFRvIII antigen was not expressed on recurrent tumors in 20/23 patients who had been initially treated with the EGFRvIII vaccine [25].

The novel EGFRvIII epitope exists extracellulary and is a prime target for monoclonal antibody recognition [118, 119], which stimulates antitumor cytotoxic T cell maturation. EGFRvIII-specific titers are not found in normal volunteers, but are present in patients with EGFRvIII-expressing cancers, such as adenocarcinomas and gliomas [120, 121]. Early animal studies using vaccination strategies against EGFRvIII reported increased numbers of tumor-infiltrating CD4+, CD8+, natural killer (NK) cells, and macrophages as well as a dramatic increase in survival [119, 122–125].

These promising preclinical results lead to early-phase I studies looking at the use of vaccine strategies against the EGFRvIII peptide. The first study for malignant gliomas was the Vaccine for Intra-Cranial Tumors I (VICTOR1). In this study, autologous mature dendritic cells were pulsed with 500 ug of PEPvIII, which was conjugated with keyhole limpet hemocyanin (KLH). Following surgical resection and completion of radiation therapy, all patients were vaccinated three times; the first three patients were dosed with 3 × 107 mature DCs per vaccine while the remaining patients were dosed with one third of their DCs per injection. No serious adverse events were reported and immunological responses were detected *ex vivo*. For patients with GBM, the median time to progression (TTP) was 46.9 weeks and median survival was 110.8 weeks. These results compare favorably with patients treated with resection site carmustine wafers [126] or temozolamide [1, 121].

The follow-up phase II study, A Complementary Trial of an Immunotherapy Against Tumor Specific EGFRvIII (ACTIVATE) evaluated the efficacy of the PEPvIII-KLH and granulocyte macrophage-colony stimulating factor (GM-CSF) [127]. Patients received three vaccinations at two-week intervals. Similar to the VICTOR1 study, there were no serious adverse effects and cellular immune responses were detected *ex vivo*. The median TTP was 14.2 months and the median survival was 32 months. Of note, upon histological examination, recurrent tumors did not express EGFRvIII. 

The currently ongoing ACTIVATE II trial was initiated to evaluate the effectiveness of adding adjuvant PEPvIII-KLH vaccination therapy to standard of care (resection, temozolomide, and radiation). Of note, temozolomide induces lymphopenia, theoretically decreasing the efficacy of an immune-based therapy. Therefore, the EGFRvIII vaccine (CDX-110) was given on day 21 of the 28 day cycle, allowing recovery of the immunosuppression caused by temozolamide [128].

#### 2.3.2. IL-13 Receptor *α*2

The IL-13R*α*2 antigen is a promising target for immunotherapy because it is highly expressed on glioma cells but not on host CNS cells [129, 130]. However, it should be noted that IL13R*α*2 expression is often heterogeneous [131]. In a study by Okano et al., it was shown that a novel epitope of IL-13R*α*2 induced CD8+ T cells to secrete IFN*γ* and lyse IL-13R*α*2-expressing glioma cells *in vitro*. This effect was only seen in CD8+ T cells expressing the HLA-A*0201 allele [132], which 40–50% of Caucasians and Asians express [133]. To target the IL-13R*α*2 in vivo, IL-13 was tagged with a mutated form of the pseudomonas exotoxin [134–138]. This fused protein (IL-13-PE38QQR), also termed Cintredekin besudotox (CB), showed promise *in vivo*; Kawakami et al. reported that CB injected intracranially resulted in both tumor regression and prolonged survival by 164% as compared with control animals [139]. 

Three phase I studies were undertaken to determine the safety of intracerebral administration of CB. Pooled results of the 51 total patients indicated a slight survival advantage as compared with BCNU wafers. Subsequently, 276 patients were enrolled in a Phase III study (PRECISE) to determine if the overall survival, safety, and quality of life differ in patients receiving the CB via local Convection-enhanced delivery (CED) compared to patients receiving BCNU wafers. There was no reported difference in median survival (36.4 weeks for the patients receiving CB compared with 35.3 weeks for the patients receiving Gliadel wafers, *P* = 0.476) [140, 141].

#### 2.3.3. IL-4 Receptor

IL-4 receptor (IL-4R) is an attractive target for immunotherapy because tumor cells express a different IL-4R isoform than that which is present on circulating immune cells. This isoform of the IL-4R is commonly expressed in human gliomas and not on neural tissue [178–181]. The type 2 IL-4R signals through the Jak-STAT pathway, activating the Jak1/Jak2 tyrosine kinases, and eventually activating the STAT-6 protein, which translocates to the nucleus and regulates gene expression [182–184]. To target the IL-4R, IL-4 was fused to pseudomonas exotoxin (IL-4(38-37)-PE38KDEL) [181, 185]. Joshi et al. showed that this construct induces glioma cell death in culture [186]. *In vivo* studies demonstrated the same construct decreased the size of implanted human-derived glioma tumors (U251) with all treated mice showing complete regression. The tumors recurred in 50% of animals but were smaller than tumors harbored by control animals [187]. 

A phase I clinical trial of the IL-4-fused protein (cpIL4-PE) was performed in patients with recurrent malignant gliomas. The construct was injected intratumorally by CED. The authors concluded that direct glioma injection of cpIL4-PE was safe, had no systemic toxicity, and caused necrosis of malignant gliomas that were refractory to conventional therapy. Subsequent clinical trials using the same construct, with stereotactic injection as the delivery method, showed similar findings of safety and efficacy [142]. 

**Figure 2:**
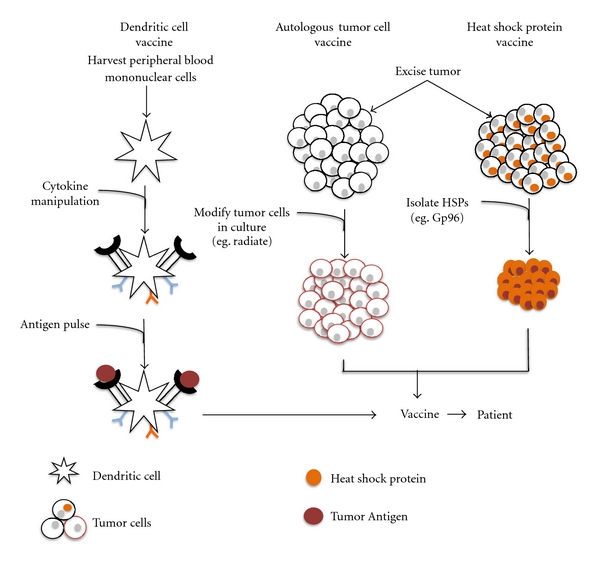
Vaccine Strategies for GBM. (From Left) *Dendritic cell vaccine:* peripheral blood mononuclear cells are isolated from the patient and cultured *ex vivo*. Cytokines are added to culture to activate the DCs. The matured DCs are pulsed with tumor antigen and then added to the vaccine preparation. *Autologous tumor cell vaccine:* after tumor removal, tumor cells are cultured. In some cases, these cells are modified (e.g., radiation, chemical) and then injected back into the patient. *Heat shock protein vaccine:* after tumor removal, tumor cells are cultured and specific heat shock proteins (e.g., Gp96) are isolated and purified. The proteins are then added to the vaccine preparation and injected into the patient.

In addition to identifying appropriate epitopes, an effective immunotherapy strategy must be able to efficiently target these antigens *in vivo*. Dendritic cell, autologous tumor cell, and heat shock protein vaccines are discussed below with general principles illustrated in Figure 2.

#### 2.3.4. Dendritic Cells

Dendritic cells (DCs) are “professional” antigen-presenting cells (APCs) that activate innate and adaptive immune responses [155]. Strategies using DCs seek to exploit this ability as GBM cells are unable to reliably present antigens to the immune system [188, 189]. DCs can be harvested from peripheral blood or bone marrow, pulsed with tumor lysate or tumor-specific peptides, and after maturation, injected back into the patient. 

In a phase I trial, Yu et al. expanded peripheral blood cells *ex vivo *into DCs and pulsed them with peptides eluted from the surface of cultured autologous brain tumor cells. Seven patients received three biweekly intradermal vaccinations of peptide-pulsed DCs with no systemic side effects. The vaccination led to significant T-cell-specific cytotoxicity against glioma tumor cells and later biopsy showed that cytotoxic and memory T cells were able to traffic into the tumor [155]. Liau et al. reported a series of 12 patients treated with 1, 5, and 10 million autologous dendritic cells pulsed with autologous tumor peptides. Similar to the previous studies, no systemic side effects were seen and survival was improved compared to historical controls. Of note, the magnitude of the T cell infiltration was inversely correlated with TGF-*β* expression within the tumor microenvironment [156]. A larger trial showed 8 of 19 patients with GBM had a median survival of 33.6 months with a median time to progression of 18.1 months, surpassing that of historical controls receiving standard of care. Of note, 42% of patients have survived longer than 4 years [190].

Pulsing DCs with whole tumor lysate increases the number of targeted epitopes and prevent antigen-loss escape and immune editing [191]. Parajuli et al. reported that DCs pulsed with apoptotic tumor cells or total tumor RNA led to a more robust immune response compared to DCs pulsed with tumor cells or fused with glioma cells [192]. Clinical trials using dendritic cells are summarized in Table 4.

#### 2.3.5. Autologous Tumor Cells

The use of autologous tumor cells (ATCs) as an immunotherapeutic approach has garnered attention due to the ability to activate the immune system with an increased number of potential glioma antigens. Several strategies for ATC vaccines have been tested including using irradiated glioma cells that were either autologous or allogenic. The autologous strategy was more beneficial in providing the most relevant antigens to the patient's tumor [193–195]. Recent clinical trials have shown this method can be used without systemic side effects. Schneider et al. reported 11 patients who received an autologous tumor vaccine with cells modified with Newcastle-Disease-Virus after surgery and radiation. Survival was no different compared to patients receiving surgery, radiation, and chemotherapy. No side effects were seen with the vaccine group [174]. A similar trial by Steiner et al. reported 23 patients who underwent surgery, radiation, and vaccination. There was a statistically significant increase in median progression-free survival (40 weeks versus 26 weeks in controls) and median overall survival of vaccinated patients (100 weeks versus 49 weeks in controls) [175]. Using an autologous formalin-fixed tumor vaccine, which is thought to preserve the antigenicity of the tumor cells, Ishikawa et al. studied 24 patients who received surgery, and radiation, showing no adverse events [176]. Selected clinical trials using ATCs are summarized in Table 5.

#### 2.3.6. Heat Shock Proteins

Heat shock proteins (HSPs) are chaperon proteins that aid in protein folding and are implicated in mediating adaptive and innate immune responses [195]. While there are five major families of HSPs, the HSPs Grp 96, HSP 90, HSP 70, HSP 110, and HSP 170 are considered the most immunogenic [196, 197]. HSPs aid in the folding of many proteins within the cell, and, therefore, a specific target antigen is not required, thus decreasing the potential for immune editing. Furthermore, HSPs have been shown to induce human DC maturation and to activate DCs to secrete proinflammatory cytokines making this strategy an attractive option for immunotherapy.

Clinical trials using a vaccine-based HSP strategy are currently underway. In cancers, such as metastatic melanoma, colorectal carcinoma, chronic myeloid leukemia, and renal cell carcinoma, HSP vaccines have been shown to be safe and associated with increased survival [198–201]. Parsa et al. reported a study in 12 patients with recurrent GBM, seven of the eight patients treated had a median survival time of 10.5 months compared to historical controls' median of 6.5 months [177]. Currently, two phase I/II clinical trials using the Grp 96 vaccine strategy are underway (NCT00293423, NCT00905060). Selected clinical trials using HSPs are summarized in Table 6.

## 3. Challenges in the Tumor Microenvironment

### 3.1. Cell Populations

GBM-mediated immunosuppresion arises from coordinated interactions among the diverse cell populations, cytokines, and extracellular matrix proteins in the tumor microenvironment. The nature of these interactions is yet to be fully characterized, but is likely to be more complex than initially appreciated. For example, it has been shown that 20–90% of endothelial cells in GBM-associated vasculature harbor the same mutations as the tumor cells [202] and that a subpopulation of CD133+ tumor stem cells expresses vascular-endothelial cadherin (CD144) [203]. Taken together, these findings indicate that a significant number of GBM-associated endothelial cells may arise from tumor stem cells [204]. In addition, experiences with conventional therapies have highlighted how specific cell populations give rise to resistance. For example, tumor stem cells are largely radioresistant. A recent study by Tamura et al. found that tumors in a cohort of patients with recurrent grade III and IV gliomas following treatment with radiosurgery and external beam radiation therapy were significantly enriched for CD133+ cells [205]. Interestingly, additional cell populations have been implicated in this phenomenon as well. *In vitro* studies of GBM stem cell sensitivity have not clearly demonstrated that these cells are more radioresistant than CD133− tumor cells [206]. Based on these findings, Calabrese et al. have proposed that the resistance of glioma stem cells to radiotherapy may arise from interactions within the GBM microenvironment [207]. Supporting this theory is the observation that GBM stem cells tend to reside within perivascular niches, where interactions with endothelial cells appear to impart tumor stem cell radioresistance [204, 208]. Other lines of evidence indicate that extracellular matrix proteins and hypoxia within the tumor microenvironment may impart radioresistance in tumor stem cells. These two examples illustrate the fact that an effective immunotherapy must not only target tumor cells, but must also disrupt the immunosuppressive activities of a variety of cell populations in the tumor microenvironment. 

### 3.2. Cytokines 

GBM cell lines have long been known to express high levels of immunosuppressive cytokines [209]. However, our understanding of the origins of these cytokines and the roles they play in the tumor microenvironment represents one of the most significant challenges to cytokine-based therapies for GBM. A recent study by Rodriques et al. demonstrated that expression of IL-10, TGF-*β*, and B7-H1 is induced in normal human monocytes after exposure to GBM cells [37]. TGF-*β* has also been implicated in the transformation of vascular endothelial cells to a proangiogenic phenotype characteristically associated with GBM [46]. Other studies indicate that TGF-*β* and IL-10 are more highly expressed in CD133+ than in CD133− glioma cells and that elevated expression of these cytokines specifically within tumor stem cell population correlates with a poorer prognosis [45, 210]. In order to fully understand the relationship between specific cytokines and the variety of cell populations present in the GBM microenvironment, subclassification of these cell populations may be necessary. For example, it has been suggested that the level of TGF-*β* expression as well as the effects of TGF-*β* signaling may vary among cancer stem cell subtypes [211]. Another recent study has shown that exposing GBM cells to IFN-*γ* decreased TGF-*β* but increased expression of PD-1 ligand and Indoleamine-2,3-Dioxygenase (IDO) [212]. It is reasonable to speculate that other immunosuppressive cytokines exhibit comparably complex interactions.

## 4. Therapies Directed at the Immune Microenvironment

### 4.1. STAT3 Blockade

STAT3 is a member of the signal transducer and activator of transcription (STAT) family of transcription factors. The detailed activities of STAT3 in cancer are reviewed elsewhere [213]. In brief, STAT3 is activated when Janus kinases (JAKs) phosphorylate the cytoplasmic tail of activated IL-6 family cytokine receptors [214]. The phosphorylated receptor then recruits STAT1 and STAT3 via the Src homology 2 (SH2) domain of the STAT protein [214, 215]. JAK tyrosine kinase activity subsequently phosphorylates STAT3 on Tyr 705, leading to formation of a phosphorylated-STAT3 (p-STAT3) homodimer which translocates to the nucleus and binds several promoters which regulate cytokine expression, cell differentiation, proliferation, apoptosis, and angiogenesis [216–219]. Constitutive activation of STAT3 has been implicated in the tumorigenesis of many cancers both inside and outside of the CNS and has been shown to be sufficient to transform cells to a malignant phenotype *in vitro *[220].

Some authors have reported that p-STAT3 is present in high levels in GBM cell lines [221] and in greater than 75% of tumor tissue samples [222]; however, other authors have failed to corroborate these findings [27]. In tumors exhibiting high levels of STAT3 activity, this transcription factor has emerged as a critical convergence point for many pathways known to be associated with GBM growth and invasion. In addition, increased STAT3 activation has been correlated with shorter overall survival in a cohort of patients with GBM [222].

Numerous lines of evidence indicate a protumorigenic role for STAT3 in the GBM microenvironment. STAT3 activation has been shown to be increased in GBM under hypoxic conditions, leading to elevated expression of proangiogenic factors such as vascular endothelial growth factor (VEGF) and hypoxic inducible factor-1 (HIF-1) [223]. Furthermore, STAT3 inhibition results in a reduction in endothelial cell tube formation *in vitro* [216, 223]. STAT3 has also been implicated in tumor invasion and suppression of apoptosis. For example, Chen et al. recently demonstrated that STAT3 inhibition reduces expression of the proinvasive factor matrix metalloprotease-2 (MMP-2) and the antiapoptotic factors Bcl-xL and survivin [224]. STAT3 is also critical for maintaining tumor stem cells [225]. A recent study by Villalva et al. demonstrated that siRNA knockdown or inhibition of STAT3 with the small molecule inhibitor Stattic led to decreased GBM stem cell proliferation and inhibited neurosphere formation [226]. In addition to its roles in angiogenesis, tumor invasion, apoptosis, and maintenance of tumor stem cells, STAT3 is known to act as a potent inhibitor of both innate [227] and adaptive [228] immune responses. STAT3 also induces tolerance via Treg activity, potentially through an HIF-1-mediated mechanism [229]. 

Although STAT3 has been most extensively studied as a tumor-promoting factor in GBM, evidence has recently emerged to suggest that it may act alternately as a protumorigenic factor or a tumor suppressor based on the genetic background of the tumor [230]. The theory that STAT3 may exert tumor-suppressing effects in GBM originated from the observation that STAT3 plays a prominent role in astrocyte differentiation [231, 232]. Studies of STAT3−/− astrocytes have demonstrated that these cells exhibit increased proliferation and invasion, although this mutation is not sufficient to produce malignancy [230]. In addition, STAT3 suppresses malignant transformation of astrocytes deficient in PTEN in an orthotopic transplant model in SCID mice [230] and a correlation between PTEN mutation and low levels of STAT3 activity has also been reported in human GBMs [233]. Conversely, STAT3 appears to be protumorigenic in EGFRvIII-expressing tumors [230]. The details of STAT3's interaction with EGFRvIII are currently unknown; however, evidence from breast cancer cell lines suggests that EGFRvIII may translocate to the nucleus and alter the binding of STAT3 to DNA [234]. 

The multiplicity of pro-oncogenic effects ascribed to STAT3 makes this transcription factor an attractive target for immunotherapy. Strategies to block STAT3 in GBM have focused primarily on direct inhibition using RNA interference and small molecule inhibitors or indirect inhibition by targeting upstream kinases or regulatory SOCS proteins [221, 235–237]. Although STAT3 inhibition has yielded promising results *in vitro*, applying this approach to animal models of GBM has produced mixed outcomes. In light of the finding that STAT3 may be alternately protumorigenic or suppressive to tumor growth, additional research is needed to elucidate the role of STAT3 in a variety of genetic contexts, including the background genotype of the host. 

Even if the correct patients are identified, the tumor microenvironment may pose a number of additional challenges to effective GBM therapy with STAT3 blockade. For example, although inhibiting STAT3 may overcome some of the immunosuppressive mechanisms employed by GBM, immune cells must still efficiently identify appropriate tumor-specific antigens in order to avoid immune editing. In addition, evidence has already emerged to suggest that cancer stem cells express a different immunosuppressive cytokine profile in response to STAT3 blockade than bulk tumor cells [238]. This finding highlights the principle that it will be critical to consider the effects of STAT3 inhibition on cytokine expression and signaling in the variety of cell populations present in the GBM microenvironment individually as well as in aggregate. Even if STAT3 inhibition results in generation of an antitumor immune response, this activity may be thwarted by activation of immune checkpoints such as PD-1 [29] and CTLA-4 [26]. Other barriers to STAT3 inhibition in the treatment of brain tumors include identifying small molecule inhibitors that can either cross the blood-brain barrier or be delivered locally. Nevertheless, STAT3 remains one of the most promising targets in immunotherapy for GBM and at least one small molecule inhibitor, WP1066, is currently in preclinical development. 

### 4.2. Regulatory T Cell Depletion

Tregs are a CD25+, FoxP3+ subset of CD4+ helper T cells which suppress immune activation through interactions with T cells, B cells, NK cells, DCs, and macrophages [239–243]. Tregs have been shown to express CTLA-4, to decrease the secretion of IL-2 and IFN-*γ* [244], and to skew the immune response away from a cytotoxic Th1-mediated response in favor of a Th2 response [245]. Studies of human GBM tissue samples have reported tumor-infiltrating lymphocyte populations significantly enriched for Tregs [26]. GBM cells also appear to secrete high levels of CCL22 and CCL2, which facilitates Treg trafficking to the tumor [246]. In addition, high-grade gliomas have been reported to exhibit a higher density of Tregs than low-grade tumors [247]. These observations have led to interest in developing immunotherapies for GBM that target Tregs. 

Tregs have been shown to be associated with a number of other known immunomodulatory pathways [248]. For example, the STAT3 inhibitor WP1066 has been shown to decrease Treg proliferation. In addition, CTLA-4 blockade may abrogate the immunosuppressive effects of Tregs in the tumor microenvironment without directly inhibiting their immunosuppressive properties [249–251]. Direct inhibition of Tregs is also possible with anti-CD25 antibodies and has been shown to improve survival in mouse glioma models [252]. A number of other approaches have also been proposed to inhibit Tregs in gliomas. These approaches are reviewed in detail elsewhere [253]. 

Indirect evidence for the efficacy of Treg depletion in human glioma comes from combining immunotherapy with cyclophosphamide, which preferentially inhibits Treg activity at low doses [254]. Clinical trials combining cyclophosphamide with a dendritic cell vaccine for renal cell carcinoma [255] or with a protein antigen vaccine for breast cancer [256] have demonstrated that the addition of cyclophosphamide augmented the antitumor effect. Blocking antibodies against CTLA-4 [249] and CD25 [252] have been shown to be effective against gliomas in mice; however, neither of these approaches has been evaluated in clinical trials.

One of the primary challenges impeding the development and implementation of Treg depletion for treatment of GBM is precisely delineating how these cells interact with the other immunosuppressive factors in the tumor environment. Despite numerous lines of evidence implicating a protumorigenic role for Tregs, and the theoretical appeal of these cells as targets for immunotherapy, fundamental questions about the role of Tregs in GBM tumorigenesis remain unanswered. For example, several studies have failed to convincingly correlate the density of tumor-infiltrating lymphocytes with prognosis in human gliomas [257–259]. Because these studies did not account for lymphocyte activity, it has been proposed that local immunosuppression in GBMs results from inhibition of T cell function secondary to an enriched population of Tregs [247]. Studies directly evaluating the relationship between Treg fractions and survival in patients with GBM, however, have not demonstrated a reliable correlation [260]. 

Tregs have been implicated in association with many other known immunosuppressive factors in the GBM microenvironment, such as CTLA-4 and STAT3. The lack of a clearly defined mechanism underlying the interactions between Tregs and CTLA-4, however, precludes the development of maximally effective combination therapies. The finding that STAT3 blockade inhibits Treg function is intriguing and deserves further exploration. In particular, STAT3 signaling may coordinate the activities of Tregs with other cell populations in the tumor microenvironment, including tumor stem cells [238]. Ultimately, defining the roles of Tregs in GBM represents a critical step toward understanding the mechanisms underlying the immunosuppressive tumor microenvironment and may serve as a valuable target for intervention. 

## 5. Conclusion

We have reviewed challenges presented by the tumor microenvironment and many of the current approaches to immunotherapy for GBM. It is becoming increasingly clear that GBM-mediated immunosuppression arises not only from the intrinsic properties of tumor cells, but from the ability of these cells to coordinate the activities of a diverse set of cell types and signaling pathways in the tumor microenvironment. Therefore, the development of effective immunotherapies will require careful study of how intervening at any point in this system alters the dynamics of these interactions. For example, the finding that treatment with IFN-*γ* increases expression of PD-L1 demonstrates potentially redundant immunosuppressive mechanisms. The differential effects of STAT3 blockade based on tumor genetics highlights the importance of developing molecular classification schemes that reflect responsiveness to various immunotherapy approaches. Furthermore, the finding that tumor stem cells may differentiate into vascular endothelial cells suggests potential interactions between tumor endothelial cells and immune cells that have not yet been elucidated. With these challenges, however, comes enormous potential to precisely target the defense mechanisms in GBM and tip the balance back in favor of the immune system.

## Figures and Tables

**Table 1:** Selected clinical trials using cytokine modulation.

Reference	Patients	Cytokine	Immunologic response	Clinical response
[58]	*n* = 145 (GBM: 103, AA: 42)	TGF-*β*	—	Median survival: 39.1 mo (10 uM dose) and 35.2 mo (80 uM dose)
[63]	*n* = 9 Recurrent GBM	IL–2	—	Enhancement of tumor on MRI unchanged (6/9)
[64]	*n* = 9 (GBM: 7, AA: 2)	IL–2	—	PR: 1
[65]	*n* = 12 recurrent GBM	IL–2	Increased inflammatory infiltrate in biopsied tumors	PR: 2, SD: 4, Minor response: 4, Overall survival 58% (6 mo) and 25% (1 yr)
[69]	*n* = 31 (GBM: 26, AA: 5)	IFN-*γ* (*n* = 14)	—	PR: 3 (Treatment group), No difference in median survival between treatment and control groups
[70]	*n* = 40 (GBM: 14, AA: 14, Other: 12)	IFN-*γ*	—	No difference in median overall survival
[71]	*n* = 29 (AA: 12, Other: 17)	IFN-*β*	—	PR: 2, SD: 2
[72]	*n* = 20 (GBM/AA: 15)	IFN-*β*	IFN-*β* treatment showed no growth suppression in *ex vivo* assays	SD: 3
[73]	*n* = 7 (GBM: 6, Recurrent AA: 1)	IFN-*β*	—	No response
[75]	*n* = 35 recurrent HGG	IFN-*α*	—	Median survival: 13.3 mo
[76]	*n* = 275 HGG	IFN-*α*	—	No difference in survival
[77]	*n* = 9 (GBM: 6, AA: 2, Other 1)	IFN-*α*	—	CR: 2
[80]	*n* = 12 (GBM: 11, AA: 1)	IL–4	Positive Elispot assay	No difference in progression free survival
[142]	*n* = 9 recurrent GBM	IL–4	—	Survival > 18 mo (*n* = 1)
[84]	*n* = 15 (GBM: 6, AA: 7, Other: 2)	IL–12	—	PR: 4, Mixed response: 1

**Table 2:** Selected clinical trials using lymphokine activated killer (LAK) cells.

Reference	Patients	Immunologic response	Clinical response
[64]	*n* = 9 (GBM: 7, AA: 2)	—	PR: 1
[91]	*n* = 9 HGG	Cultured LAK cells lysed cultured glioma cells (*n* = 6)	Slight clinical (but not radiologic) improvement.
[92]	*n* = 20 recurrent HGG	—	Median survival: 63 weeks
[93]	*n* = 19 (GBM: 5, AA:4, Other 10)	—	CR: 1, PR: 2, median survival (GBM): 15 weeks
[94]	*n* = 40 recurrent GBM	—	Median survival: 17.5 months (significantly longer than contemporary patients)
Others: [94, 143–147]			

**Table 3:** Selected clinical trials using cytotoxic T lymphocytes (CTLs).

Reference	Patients	Immunologic response	Clinical response
[97]	*n* = 4 (GBM: 3, AA: 1)	—	PR: 3
[98]	*n* = 12 (GBM: 6, AA: 1, Other: 5)	—	PR: 4
Others: [20, 97, 148–154]			

**Table 4:** Selected clinical trials using dendritic cells (DCs).

Reference	Patients	Immunologic response	Clinical response
[155]	*n* = 7 (GBM: 6, AA: 1)	Cytotixic and memory T cells found in recurrent tumor bulk	Median survival: 455 days (Control group: 257 days)
[156]	*n* = 12 (GBM: 7, Recurrent GBM: 5)	Cytotoxicity against autologous tumor cells. Cytotoxic T cells found in recurrent tumor bulk.	Median TTP: 19.9 mo (*P* = 0.028), Median survival: 35.8 mo (*P* = 0.006)
[157]	*n* = 18 EGFRvIII expressing GBM	82% of recurrent tumors lost EGFRvIII expression	Median survival: 26 mo (*P* = 0.001)
Others: [80, 84, 158–173]			

**Table 5:** Selected clinical trials using autologous tumor cells (ATCs).

Reference	Patients	Immunologic response	Clinical response
[174]	*n* = 11 recurrent GBM	Local skin reaction	Median survival: 46 weeks
[175]	*n* = 23 GBM	Delayed-type hypersensitivity, increased memory T cells, increased CD8+ T cells in recurrent tumors	Median progression free survival: 40 weeks, median survival 100 weeks
[176]	*n* = 12 GBM	—	CR: 1, PR: 1, minor response: 2, median survival: 10.7 mo

**Table 6:** Selected clinical trials using heat shock proteins (HSP).

Reference	Patients	Immunologic response	Clinical response
[177]	*n* = 12 recurrent GBM	—	Median survival: 10.5 mo

## Abbreviations

CR: Complete response
PR: Partial response
SD: Stable disease
TTP: Time to progression
GBM: Glioblastoma multiforme
AA: Anaplastic astrocytoma.
